# Assisted reproductive techniques with congenital hypogonadotropic hypogonadism patients: a systematic review and meta-analysis

**DOI:** 10.1186/s12902-018-0313-8

**Published:** 2018-11-19

**Authors:** Yinjie Gao, Bingqing Yu, Jiangfeng Mao, Xi Wang, Min Nie, Xueyan Wu

**Affiliations:** Key laboratory of Endocrinology, Ministry of Health; Department of Endocrinology, Peking Union Medical College Hospital, Peking Union Medical College, Chinese Academy of Medical Sciences, Wang Fu Jing St, Dongcheng district, Beijing, 100730 China

**Keywords:** Congenital hypogonadotropic hypogonadism, Assisted reproductive techniques, Fertility, Hormonal replacement therapy

## Abstract

**Background:**

After hormonal replacement therapy (HRT) including androgen replacement or sequential therapy of estrogen and progesterone, The combination of human chorionic gonadotropin (hCG) and human menopausal gonadotropin (hMG) and pulsatile GnRH, is not sufficient to produce sufficient gametes in some patients with Congenital hypogonadotropic hypogonadism (CHH). A Systematic review and meta-analysis was performed to determine that assisted reproductive techniques (ART) can effectively treat different causes of infertility.

**Methods:**

To determine the effect of ART on fertility of CHH patients and investigate whether outcomes are similar to infertility due to other causes, we conducted a systematic review and meta-analysis of retrospective trials.

Clinical trials were systematically searched in Medline, Embase, and the Cochrane central register of controlled trials databases. The keywords and major terms covered “hypogonadotropic hypogonadism”, “kallmann syndrome”, “assisted reproductive techniques”, “intrauterine insemination”, “intracytoplasmic sperm injection”, “testicular sperm extraction”, “in vitro fertilization”, “embryo transplantation” and “intra-Fallopian transfer”.

**Results:**

A total of 388 pregnancies occurred among 709 CHH patients who received ART (effectiveness 46, 95% confidence interval 0.39 to 0.53) in the 20 studies we included. The I^2^ in trials assessing overall pregnancy rate (PR) per embryo transfer (ET) cycle was 73.06%. Similar results were observed in subgroup analysis by different gender. Regression indicates pregnancy rate decreases with increasing age. Fertilization, implantation and live birth rates (72, 36 and 40%) showed no significant differences as compared to infertility due to other causes.

**Conclusions:**

Despite CHH patients usually being difficult to generate gametes, their actual chances of fertility are similar to subjects with other non-obstructive infertility. ART is a suitable option for CHH patients who do not conceive after long-term gonadotropin treatment.

**Electronic supplementary material:**

The online version of this article (10.1186/s12902-018-0313-8) contains supplementary material, which is available to authorized users.

## Background

Congenital hypogonadotropic hypogonadism (CHH) is a disorder characterized by lacking of puberty and infertility, with low levels of circulating gonadotropins and sex steroids. Two pathogenesis mechanisms exist for CHH. One is the reduced secretion of gonadotropin releasing hormone (GnRH) from the hypothalamus, and the other is the GnRH receptor defect in the pituitary. The incidence of CHH is approximately 1/10000–1/86000 [1], and the ratio of male versus female is about 3.6–1 which varies from race to race [2]. About 50% cases who show anosmia/hyponosmia simultaneously called Kallmann syndrome [3]. The Genetic defects are the main underlying mechanism. More than 30 pathogenic genes of CHH have been identified [4].

Therapy for CHH depends on the patients’ desire for fertility at the time of treatment. Androgen replacement or sequential therapy of estrogen and progesterone can be used for patients who do not wish to have children. The combination of human chorionic gonadotropin (hCG) and human menopausal gonadotropin (hMG) is used to induce fertility. Pulsatile GnRH is another option for CHH patients who desire a pregnancy.

Nevertheless, the treatment for inducing fertility may not be effective for all CHH patients. Alternate fertility inducing methods have been described for patients who do not respond to hormone replacement therapy (HRT) as HRT replaces lacking hormones rather than inducing ovulation or spermatogenesis. Different assisted reproductive techniques (ART) can improve conception rate. Intrauterine insemination (IUI) is suitable for patients with sexual dysfunction and obstructive fertility. In vitro fertilization-embryo transplantation (IVF-ET) is appropriate for infertility due to many causes, especially the disorder of sperm-egg binding. Intracytoplasmic sperm injection (ICSI) and testicular sperm extraction (TESE) are used for infertility due to decreased quality and quantity of sperms. ART might be an efficient approach to treat infertility in CHH patients due to various causes. The aim of this review was to meta-analyze the pregnancy outcomes in order to reveal the effect of ART on CHH patients, and whether it is distinct from infertility due to other causes.

## Methods

This systematic review and meta-analysis was conducted following the Preferred Reporting Items for Systematic Review and Meta-Analyses (PRISMA) statement [5] (Additional file 1: Table S1). Moreover, the analyses were based on previous published studies, thus no ethical approval and patient consent are required according to the regulation of Peking Union Medical College Hospital ethic committee. All previous published studies were approved by ethics committee respectively.

### Data sources and searches.

An electronic search of Medline, Embase, and the Cochrane central register of controlled trials was performed. We used keywords and major terms including “hypogonadotropic hypogonadism”, “kallmann syndrome”, “assisted reproductive techniques”, “intrauterine insemination”, “intracytoplasmic sperm injection”, “testicular sperm extraction”, “in vitro fertilization”, “embryo transplantation” and “intra-Fallopian transfer”.

There were no language restrictions, and the retrieval was till March 2018. The detailed search strategies are shown in Additional file 2: Table S2.

### Study selection

The included studies were retrospective and researched the positive effect of ART treatment on CHH patients who did not achieve pregnancy after HRT. The inclusion criteria were trials that evaluated at least one of the primary or secondary outcomes mentioned below. The primary outcomes included several pregnancy-related indicators, including fertilization rate, implantation rate, clinical pregnancy per cycle or embryo transfer (ET), and live birth rate. Secondary outcomes included the comparison of pregnancy rates according to different genders, some adverse events arising from ART, such as abortion, multiple gestation and ovarian hyperstimulation syndrome (OHSS). In addition, all the 20 studies we included have clear patient inclusion criteria. The hypophyseal axis was checked by measuring TSH, cortisol, and prolactin, which showed no other combined pituitary hormone deficiency.

Exclusion criteria were [1]: klinefelter syndrome [2]; adult-onset (secondary) HH [3]; reviews and case reports [4]; no available end-points; and [5] duplications or sub-studies of the included trials.

### Data extraction and quality assessment

All studies included in the meta-analysis were reviewed and data on author, year of publication, study design, time, location, number, age and gender of subjects, the duration and methods of HRT, type of surgery and number of cycles were extracted. In addition, fresh/frozen sperm and spouse’s age in males, and body mass index (BMI) in females were observed. The end-points after ART were also extracted for analysis, including fertilization rate, implantation rate, clinical pregnancies, clinical pregnancy per cycle or ET, live birth children, live birth rate and total number of adverse events reported (Table 1).

**Table 1:** Information of selected studies

Author, year	No.pts	Age mean/SD (year)	BMI mean/SD (kg/m^2^)	Treatment, duration	Control group	Type of surgical, No. of cycles	Fresh/frozen sperm	Spouse’s age (year)	Outcomes (PR, FR, IR) and final outcome (LBR)	Adverse events
Female										
Ulug 2005	58	32.2/5.2	21.09/1.3	HCG, HMG,	TI	IUI, IVF (ICSI)-ET, 53 cycles	n/a	n/a	PR 56.6%,	abortion,
				14 days	*n* = 16				FR 73.9%,	multiple pregnancy
									IR 32.4%	
Kumbak 2006	27	32.8/4.9	25.7/4.5	gonadotrophins,	UI	IVF (ICSI)-ET,	n/a	n/a	PR 59.3%,	multiple pregnancy
				14 days	*n* = 39	27 cycles			FR 89%,	
									IR 36.5%	
Yildirim 2010	13	31.3/5.6	25.3/3.1	gonadotrophins,	TI	IVF (ICSI)-ET,	n/a	n/a	PR 80%,	abortion
				13 days	*n* = 20	13 cycles			FR 81.9%,	
									IR 38.3%,	
									LBR 50%	
Dokuzeylul 2010	57	≤37	n/a	gonadotrophins,	normal control	IVF (ICSI)-ET,	n/a	n/a	PR 56.14%,	n/a
				14 days	*n* = 95	57 cycles			IR 36.7%	
Caragia 2012	17	32.96/3.976	n/a	FSH, LH,	TI/MI/UI	IVF (ICSI)-ET,	n/a	n/a	PR 55.6%,	no adverse events
				16 days	*N* = 56/56/56	28 cycles			FR 55.4%,	
									LBR 54%	
Ghaffari 2013	81	33.5/5.3	26.1/4.0	HMG, HCG, P, E2,	TI	IVF (ICSI)-ET,	n/a	n/a	PR 19.4%,	abortion,
				14 days	*n* = 89	72 cycles			FR 61.2%,	multiple pregnancy
									IR 40%,	
									LBR 15.2%	
Pandurangi 2015	7	27	25.29/3.77	HMG, uFSH,	n/a	IUI, IVF (ICSI)-ET, 19 cycles	n/a	n/a	PR 31.6%,	abortion,
				29 days					FR 85%,	multiple pregnancy
									LBR 85.7%	
Yilmaz 2015	33	32.5/4.73	26/ 3.81	HMG, HCG, recFSH, P,12 days	MI	IVF (ICSI)-ET,	n/a	n/a	PR 30%	n/a
					*n* = 47	33 cycles				
Jiang 2017	46	30.9/3.9	21.26/1.89	HCG, HMG,	TI	IUI, IVF (ICSI)-ET, 42 cycles	n/a	n/a	PR 59.52%,	abortion,
				13 days	*n* = 71				FR 82.13%,	multiple pregnancy
									IR 41.46%	
Mumusoglu 2017	57	30.6/5.1	25.6/4.5	FSH,	TI	IVF (ICSI)-ET,	n/a	n/a	PR 36.8%,	abortion
				11 days	*n* = 114	57 cycles			IR 34.4%,	
									LBR 36.8%	
Kuroda 2018	79	32.6/0.5	18/0.3	HCG, HMG,	n/a	IVF (ICSI)-ET,	n/a	n/a	PR 59.3%,	abortion
				12.2 days		117 cycles			LBR 45.9%	
Male										
Fahmy 2004	15	38.71/6.2	n/a	HCG, HMG,	n/a	TESE+IVF (ICSI)-ET,	n/a	n/a	PR 16.7%,	multiple pregnancy
				6 months		17 cycles			FR 41.7%	
Zorn 2005	4	37.75	n/a	HCG, HMG,	n/a	IVF (ICSI)-ET,	fresh/	31.5	PR 33.3%,	no adverse events
				13.25 months		9 cycles	frozen sperm		IR 21%	
Bakircioglu 2007	25	34.5/5.2	n/a	HCG,	n/a	TESE+IVF (ICSI)-ET,	fresh/	n/a	PR 54.5%	abortion
				9.96 months		22 cycles	frozen sperm			
Akarsu 2009	4	36.25	n/a	HCG, HMG,	n/a	TESE+IVF (ICSI)-ET,	fresh/	29.5	PR 16.7%,	abortion,
				9.96 months		18 cycles	frozen sperm		FR 41.8%,	multiple pregnancy
									LBR 75%	
Resorlu 2009	17	30.1	n/a	HCG, FSH,	n/a	IVF (ICSI)-ET,	fresh	n/a	PR 54.5%	n/a
				24 months		11 cycles	sperm			
Dokuzeylul 2010	31	34.82	n/a	HCG, HMG,	n/a	TESE+IVF (ICSI)-ET,	n/a	n/a	PR 51.7%,	abortion,
				6 months		29 cycles			FR 83%,	multiple pregnancy
									LBR 41.3%	
Bakircioglu 2012	12	40.2	n/a	HCG, FSH,	n/a	TESE+IVF (ICSI)-ET,	n/a	n/a	PR 66.7%,	multiple pregnancy
				15.3 months		9 cycles			LBR 77.8%	
Sahin 2012	65	n/a	n/a	gonadotrophins,	n/a	IVF (ICSI)-ET,	n/a	n/a	PR 43.9%,	n/a
				10.2 months		57 cycles			LBR 60%	
Basar 2017	61	35.8/5.64	n/a	HCG, HMG,	n/a	TESE+IVF (ICSI)-ET,	fresh/	n/a	PR 47.1%,	n/a
				n/a		119 cycles	frozen sperm		LBR 62.3%	

*TI* = tubal factor infertility; *MI* = male factor infertility; *UI* = unexplained infertility; *PR* = pregnancy rate; *FR* = fertilization rate; *IR* = implantation rate; *LBR* = live birth rate

Study quality was examined using inclusion and exclusion criteria, definition of end-points, adequacy of follow-up, data analysis and presentation. In addition, studies were scored for quality by Methodological Index for Non-randomized Studies (MINORS). The scores of quality assessment are listed in Table 2. There are eight methodological items for non-randomized studies and four additional criteria for comparative studies. The items are scored 0 (not reported), 1 (reported but inadequate) or 2 (reported and adequate). The global ideal scores are 16 for non-comparative studies and 24 for comparative studies [6].

**Table 2:** Assessment of risk of bias by Methodological Index for Non-randomized Studies (MINORS)

	1.A clearly stated aim	2.Inclusion of consecutive patients	3.Prospective collection of date	4.Endpoints appropriate to the aim of the study	5.Unbiased assessment of the study endpoint	6.Follow-up period appropriate to the aim of the study	7.Loss to follow up less than 5%	8.Prospective calculation of the study size	9.An adequate control group	10. Contemporary groups	11.Baseline equivalence of groups	12.Adequate statistical analyses	Total
Female													
Ulug (2005)	2	2	2	2	2	0	0	2	2	2	2	2	20
Kumbak (2006)	2	2	2	2	2	1	0	2	2	2	2	2	21
Yildirim (2010)	2	0	2	2	2	0	0	2	2	2	2	2	18
Dokuzeylul (2010)	2	0	2	2	2	0	0	2	2	2	2	2	18
Caragia (2012)	2	0	1	2	2	0	0	2	2	2	2	2	17
Ghaffari (2013)	2	2	2	2	2	0	0	2	2	2	2	2	20
Yilmaz (2015)	2	2	2	2	2	0	0	2	2	2	2	2	20
Pandurangi (2015)	2	2	2	2	2	0	0	0	0	0	0	0	10
Jiang (2017)	2	2	2	2	2	0	0	2	2	2	2	2	20
Mumusoglu (2017)	2	2	2	2	2	0	0	2	2	2	2	2	20
Kuroda (2018)	2	2	2	2	2	1	0	2	0	0	0	0	13
Male													
Fahmy (2004)	2	2	1	2	2	0	0	0	0	0	0	0	9
Zorn (2005)	2	1	2	2	2	1	0	0	0	0	0	0	10
Bakircioglu (2007)	2	1	2	2	2	1	0	0	0	0	0	0	10
Akarsu (2009)	2	2	2	2	2	0	0	0	0	0	0	0	10
Resorlu (2009)	2	1	2	2	2	0	0	0	0	0	0	0	9
Dokuzeylul (2010)	2	0	1	2	2	0	0	0	0	0	0	0	7
Bakircioglu (2012)	2	1	1	2	2	0	0	0	0	0	0	0	8
Sahin (2012)	2	1	1	2	2	0	0	0	0	0	0	0	8
Basar (2017)	2	0	1	2	2	0	0	2	0	0	0	0	9

The items are scored 0 (not reported), 1 (reported but inadequate) or 2 (reported and adequate). The global maximum scores are 16 for non-comparative studies and 24 for comparative studies

### Data synthesis and statistical analysis

Data were pooled using a random effects model to obtain a more conservative estimate of ART on CHH patients who were unresponsive or not pregnant after long-term treatment with gonadotropins, allowing for any heterogeneity between studies.

Heterogeneity between studies was assessed using the I^2^ statistic with a cut-off of ≥50%, and the X^2^ test with a *p* < 0.10 to define a significant degree of heterogeneity. Where the degree of statistical heterogeneity was greater than this between trial results, possible explanations were investigated using subgroup analysis according to the gender. It can tell the possible reason of heterogeneity. These were exploratory analyses only and may explain some of the observed variability, but the results should be interpreted with caution. The 95% confidence intervals were used to generate Forest plots of pooled relative risks for primary and secondary outcomes. The sample size and risk of bias of included trials is crucial for the weighted differences, which means the more representative study is, the greater the weight is. We also performed a meta-regression analysis by age to show its influence on the pregnancy outcome.

## Results

### Literature search

**Fig. 1:**
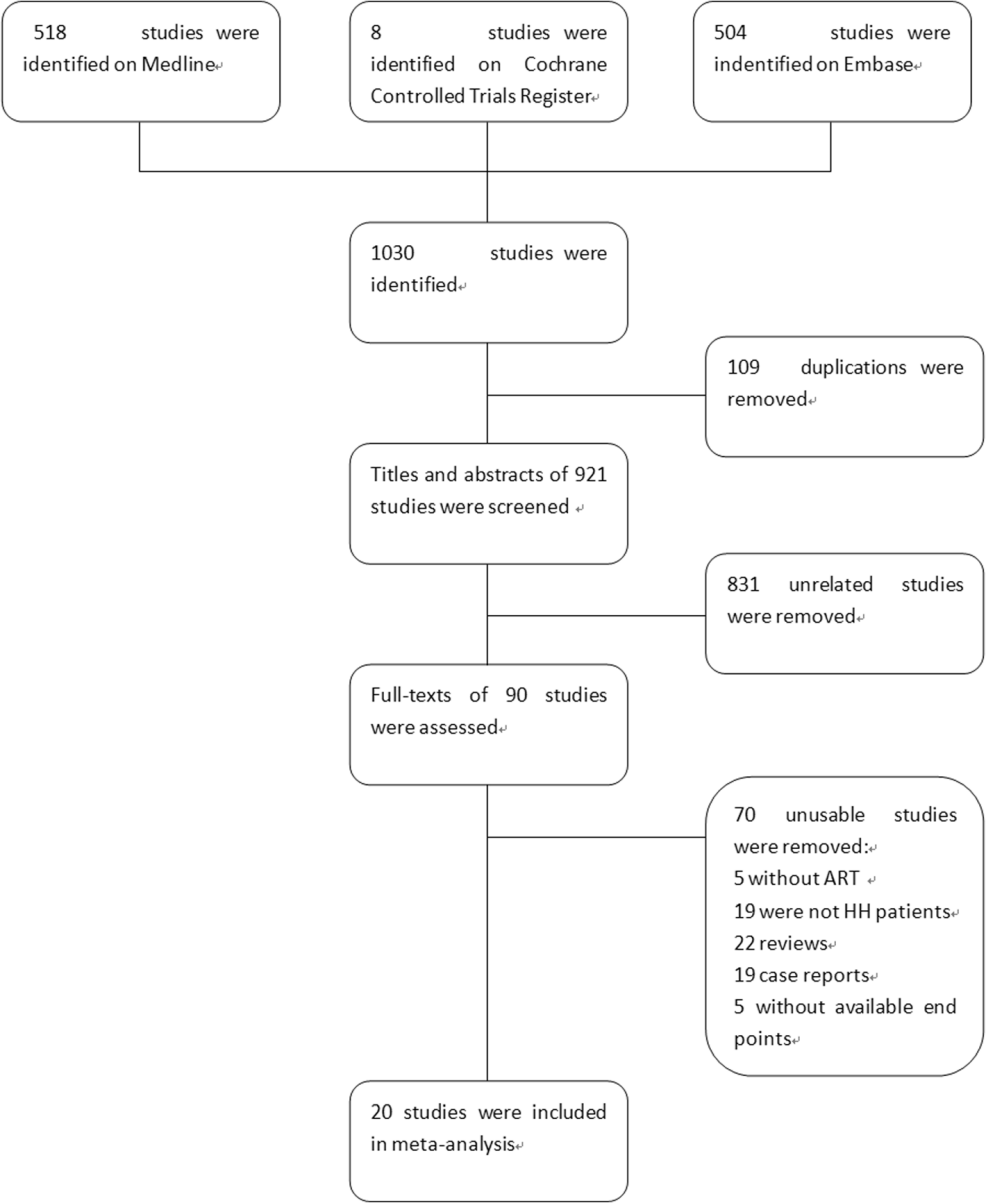
Flow diagram of studies identified in the systematic review

The search strategy identified 1030 citations. After eliminating duplicates, 921 studies were subsequently reviewed. The titles and abstracts were screened, and 90 articles were potentially eligible for inclusion. Only 20 of these were retrieved after reading the full-texts. The data in these studies could be consolidated and analyzed (Fig. 1).

### Clinical characteristics of patients in the included studies

In the 20 studies included, a total of 475 female CHH patients were included in eleven studies and 234 male CHH patients in the other nine studies. The range of average ages of female CHH patients was 27 to 33.5 years old, and 30.1 to 40.2 years old for male CHH patients. Average BMI of female patients was between 18 to 27 kg/m^2^, and no BMI values were described in studies in male patients. Nine out of eleven studies on female CHH patients contained control groups which comprised a total of 659 infertile cases caused by other reasons including tubal factor, male factor or other unexplained factors.

### Pregnancy outcomes

**Fig. 2:**
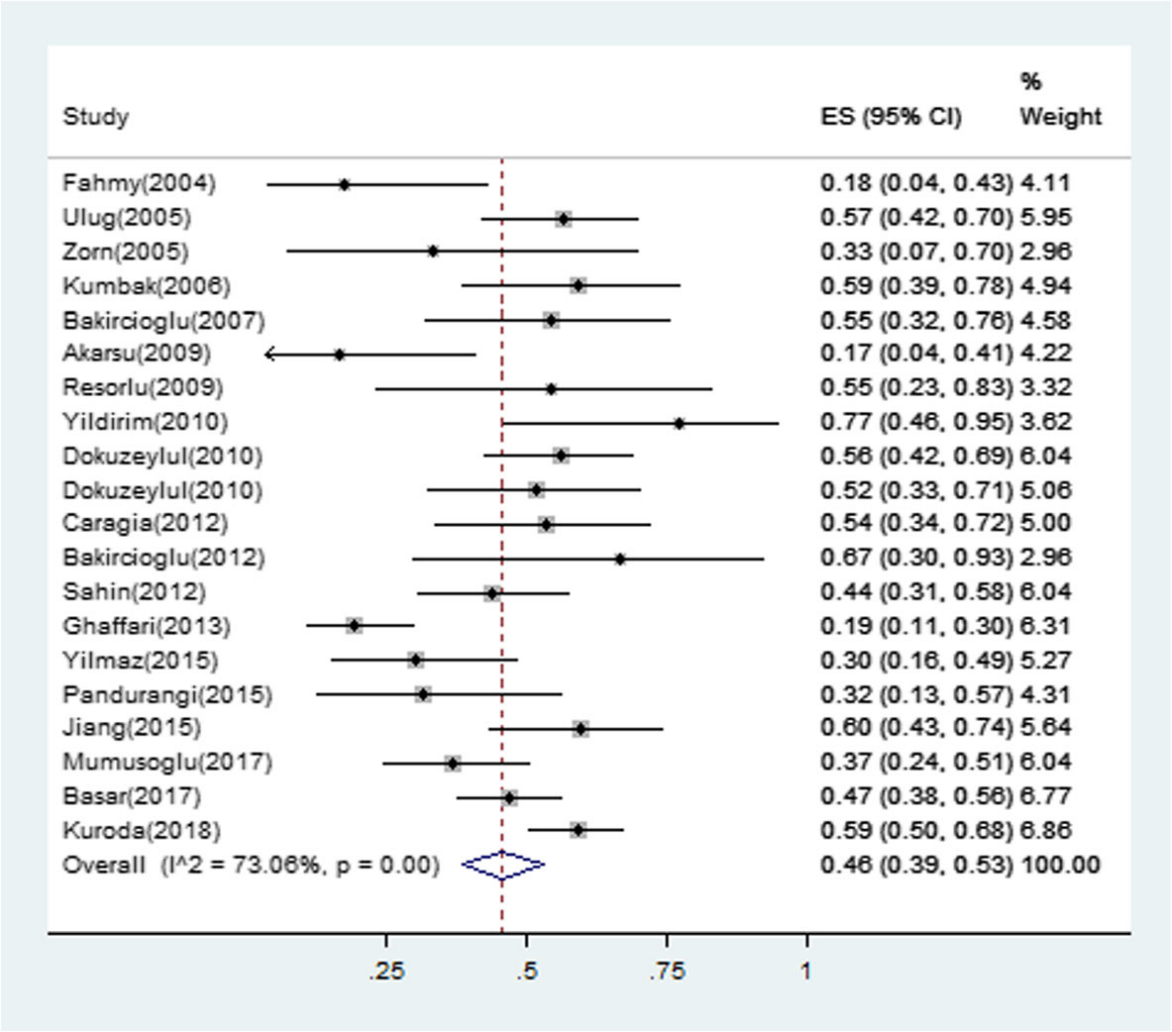
Forest plot for the pregnancy rate. ES = effect size; CI = confidence interval

**Fig. 3:**
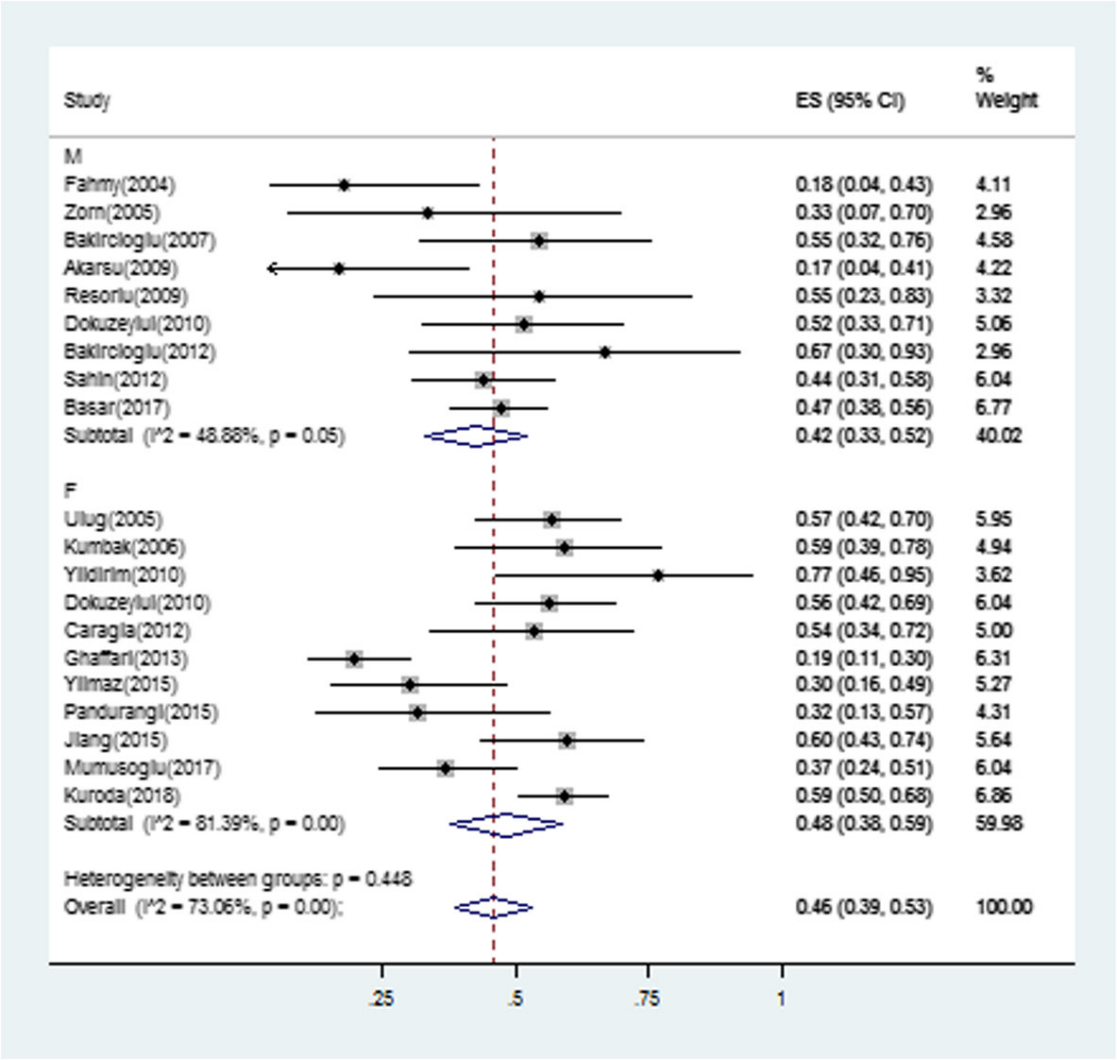
Forest plot for subgroup analysis by different gender

**Fig. 4:**
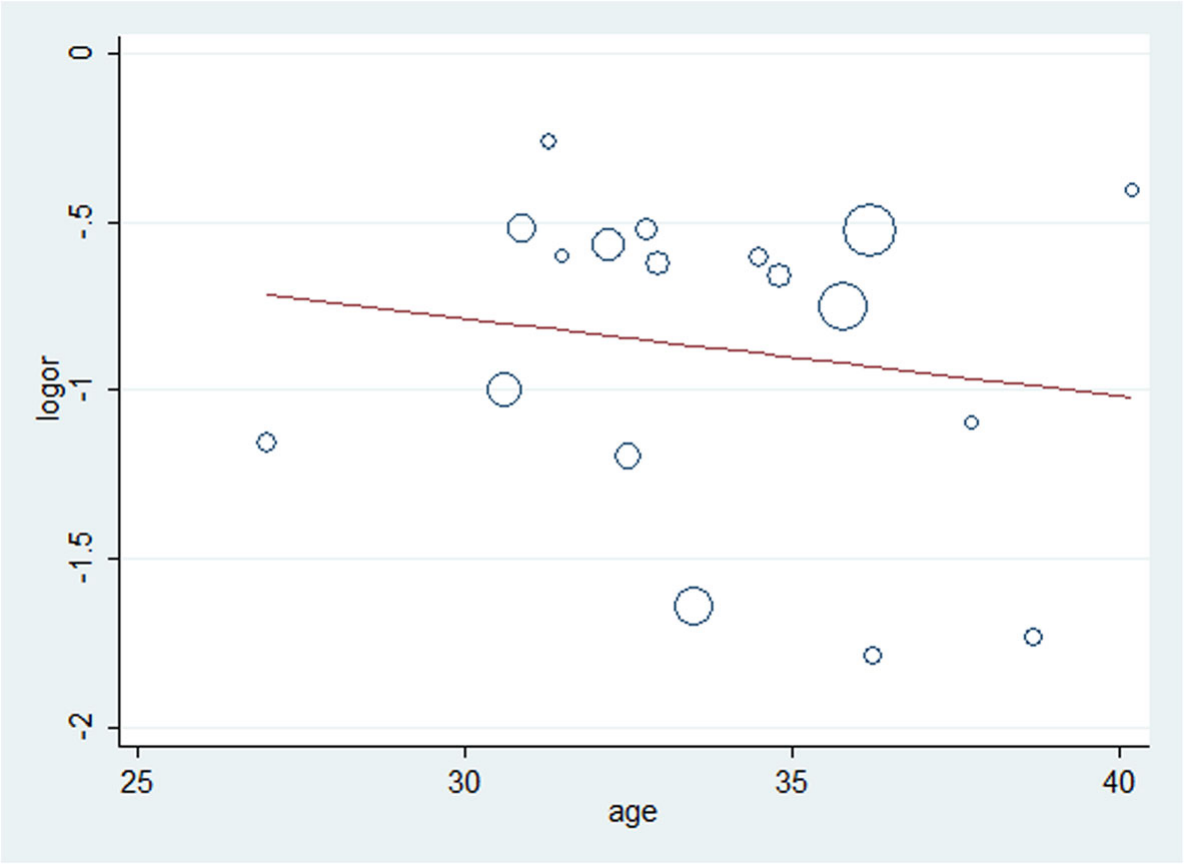
Influence of age at pregnancy rate Influence of age (X-axis) on pregnancy rate (Y-axis). The size of the circles indicates sample dimension.

The majority treatment regimens used was hCG combined with hMG, duration was around two weeks in females and 6–12 months in males. The type of ART was in vitro fertilization (IVF)/intracytoplasmic sperm injection (ICSI), 3/11 female trials used extra IUI, and 6/9 male trials used TESE. The characteristics of the retrieved trials, including parameters on trial quality, are reported in Tables 1 and 2. A total of 388 pregnancies occurred among 709 individuals who received ART (effectiveness 46, 95% confidence interval 0.39 to 0.53) (Fig. 2). The I^2^ in trials assessing overall pregnancy rate (PR) per ET cycle was 73.06%. Similar results were observed in subgroup analysis by sex (Fig. 3). Pregnancy rate was 48% in the female group and 46% in the male group. Meta-regression analysis showed that PR per ET cycle decreased with increasing age (Fig. 4). Sufficient data was not available to evaluate the influence of infertility duration, basal BMI, testis volume or sperm counts and the levels of follicle-stimulating hormone (FSH), leuteinizing hormone (LH) and testosterone (T) on treatment effect of ART.

**Fig. 5:**
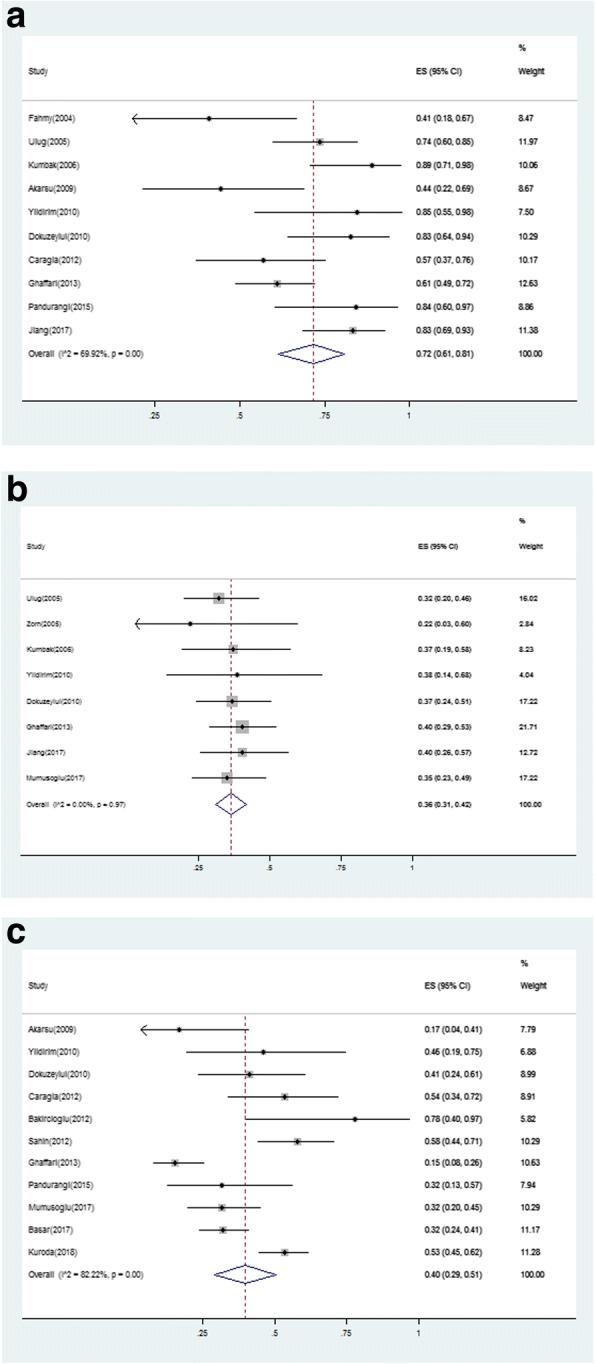
Forest plots for the fertilization rate **a**, implantation rate **b** and live birth rate (**c**)

The forest plots of fertilization rate, implantation rate and live birth rate are shown in Fig. 5. They showed no significant differences as compared to other cohorts.

### Adverse events

**Fig. 6:**
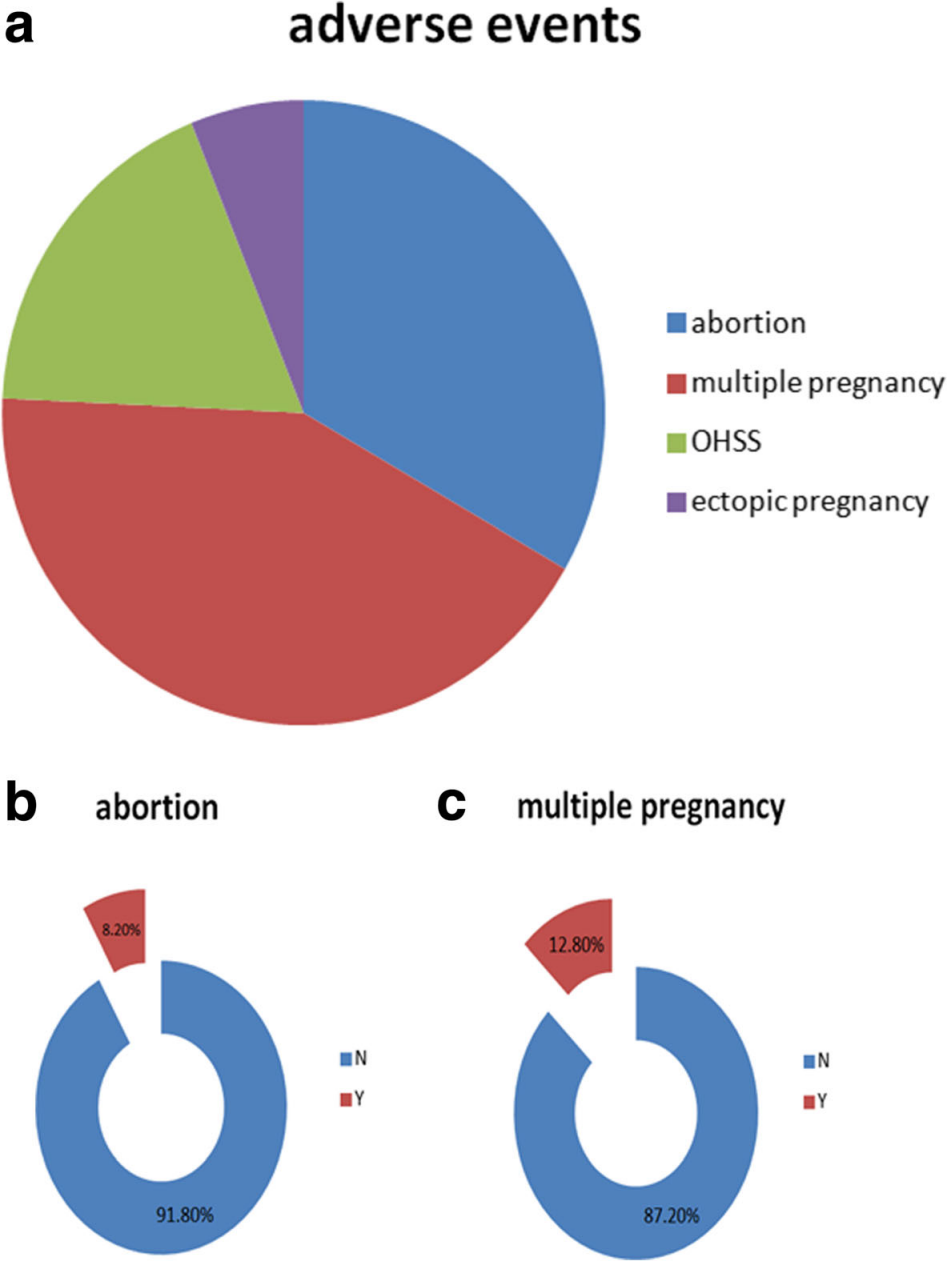
Frequencies of abortion and multiple pregnancies. (**a**)Different colors represent four adverse events, including abortion(blue),multiple pregnancy(red),OHSS(green) and ectopic pregnancy(purple);(**b**)8.20% patients of all suffered abortion(red);(**c**)12.8% patients of all had multiple pregnancy. Y=Yes; N=No

Individual adverse events were reported in many of the included studies. Fourteen out of 20 trials included abortion rate and 11/20 included the outcomes of multiple pregnancies. Six trials mentioned ovarian hyperstimulation syndrome (OHSS) but there was no OHSS occurrence in the included studies. Only two trials reported ectopic pregnancies. The frequencies of abortion and multiple pregnancies are shown in Fig. 6. There was no statistically significant difference in the incidence of adverse events for CHH patients undergoing ART as compared to infertile cohorts with other causes. Therefore, ART appears to be a safe and effective method to induce fertility in CHH patients.

## Discussion

In this study, we systematically reviewed and meta-analyzed pregnancy outcomes in CHH patients undergoing ART. For this specific population, the overall pregnancy rate per ET cycle was about 46% (95% confidence interval 0.39 to 0.53), which was comparable to the patients with other etiological infertility including tubal factor infertility (TI), male factor infertility (MI) and unexplained infertility (UI). The fertilization rate (72%), implantation rate(36%) and live birth rate(51%) were not significantly different from other cohorts. Hence, ART is a viable option for CHH patients with unsuccessful long-term HRT. However, this review had a high risk of potential bias and clinical heterogeneity caused by the study design and the inconsistency in results across the included studies. Factors such as age, BMI, sex hormone levels, pathogenesis of CHH and function of ovary in women, testis volume in men and HRT before ART may have different effects on fertility outcomes in CHH patients treated by ART, and not all these factors were analyzed in the included studies.

Two methods, including hCG combined with hMG and pulsatile GnRH, are common fertility promoting treatments for CHH patients. However, the effectiveness is around 70% [7]. About 30% male CHH patients had no or few sperm with conventional therapy (i.e. azoospermia, oligospermia). ICSI combined with TESE may improve their fertility and the pregnancy rates could be similar to those observed in other forms of infertility.

Intra-Fallopian transfer was initially applied for the spouse of a male patient with Kallmann’s syndrome [8]. Impaired semen quality prevented his spouse from conceiving and IVF helped the couple in having a healthy baby. Thereafter, some studies presented pregnancies achieved through IVF/ICSI in CHH patients not responding to hormonal treatment [9, 10], and pregnancy rates from the large studies were 50–60% [11, 12]. Gonadotropin replacement combined with TESE-ICSI cycles improved pregnancy rate of CHH patients [13], the clinical pregnancy rate was 17.6%. With the advancement of technology, various ART treatments were applied on CHH patients in different situations such as age, sex, region, duration and extent of illness. The success rate increased to 55% [14, 15]. Furthermore, a study in 1997 first emphasized that initiation of ICSI treatment after testicular maturity induced by hormonal treatment contributed to the success of ART [16].

Waiting may be advisable as maximal sperm counts are not attained until 12–18 months of treatment, and even longer in cases of cryptorchidism. However, like the general population, chances of fertility in CHH patients after ART reduced with increasing age. Quality and quantity of follicles both decreased. Older women require higher doses of gonadotropins to achieve the desired outcome due to diminished ovarian function with aging [12, 17]. Therefore, early ART for unresponsive CHH patients who received HRT for some time may be beneficial.

Multiple pregnancy, abortion, ectopic pregnancy [18] and OHSS [19] are common adverse events of ART. The multiple pregnancy and abortion rates were around 30% [20] and 14.7% [21], respectively. These rates did not increase in CHH patients who received ART therapy. Severe OHSS was not observed during ovulation induction in CHH patients [11, 22, 23]. The ovaries were dormant and needed to be stimulated with higher doses of gonadotropins, which might theoretically increase the risk of OHSS in the CHH group [24], but the frequency of OHSS was not increased in our review.

Several limitations of this meta-analysis should be emphasized. First, the number of included studies was small, which may create selective bias. Second, all included studies were retrospective. Hence, the significant statistical heterogeneities (I^2^ = 73.06% in pregnancy rate) may have influenced our findings. Third, the baseline characteristics were not described in detail, which could influence the outcomes by the confounding variables. For example, testis volume and cryptorchidism (maldescended testes) is an important indicator for sperm production, but adequate data was not available for analysis. Great progress in ART treatment also made sense to patients in different decades. Last, it seems that not all studies reported adverse events, and some like OHSS is not considered to be an adverse event, so more studies should be included to avoid the reporting bias.

## Conclusions

In summary, the present study showed that despite CHH patients usually being azoospermic, their actual chances of fertility are similar to subjects with other types of obstructive infertility. If CHH patients do not conceive after long-term gonadotropin treatment, ART should be initiated.

## Additional files

Additional file 1: Table S2. PRISMA 2009 checklist. (DOC 61 kb)

Additional file 2: Table S1. Search strategy for PubMed. (DOCX 15 kb)

## Abbreviations

ART: Assisted reproductive techniques
CHH: Congenital hypogonadotropic hypogonadism
ET: Embryo transfer
GnRH: Gonadotropin releasing hormone
hCG: Human chorionic gonadotropin
hMG: Human menopausal gonadotropin
HRT: Hormonal replacement therapy
ICSI: Intracytoplasmic sperm injection
IUI: Intrauterine insemination
IVF-ET: In vitro fertilization-embryo transplantation
OHSS: Ovarian hyperstimulation syndrome
PR: Pregnancy rate
TEST: Testicular sperm extraction
