# Supplementary material for: Assisted reproductive techniques with congenital hypogonadotropic hypogonadism patients: a systematic review and meta-analysis
Source: BMC Endocr Disord. 2018 Nov 19;18:85. doi: 10.1186/s12902-018-0313-8 (PMC6245556; doi:10.1186/s12902-018-0313-8)
Supplement: Supplementary file 2 — Search strategy for PubMed. (DOCX 15 kb) [file 12902_2018_313_MOESM2_ESM.docx]

**Table S2. Search strategy for PubMed**

#1 "Idiopathic Hypogonadotropic Hypogonadism" [Supplementary Concept] 47

#2 "Kallmann Syndrome"[Mesh] 556

#3 "Hypogonadotropic hypogonadism and anosmia, autosomal dominant" [Supplementary Concept] 1

#4 hypogonadotropic hypogonadism 16961

#5 kallmann syndrome 832

#6 （#1）OR(#2)OR(#3)OR(#4)OR(#5) 17058

#7 "Reproductive Techniques, Assisted"[Mesh] 62298

#8 "Sperm Injections, Intracytoplasmic"[Mesh] 5780

#9 "Fertilization in Vitro"[Mesh] 32430

#10 "Embryo Transfer"[Mesh] 14644

#11 assisted reproductive techniques 64105

#12 intrauterine insemination 2531

#13 intracytoplasmic sperm injection 8950

#14 testicular sperm extraction 921

#15 in vitro fertilization 42869

#16 embryo transplantation 31108

#17 intra-fallopian transfer 221

#18(#7)OR(#8)OR(#9)OR(#10)OR(#11)OR(#12)OR(#13)OR(#14)OR(#15)OR(#16)OR(#17) 86073

#19 (#6)AND(#18) 518

**Search strategy for Cochrane Controlled Trials Register**

#1 MeSH descriptor: [Hypogonadism] explode all trees 300

#2 MeSH descriptor: [Kallmann Syndrome] explode all trees 5

#3 "hypogonadotropic hypogonadism" (Word variations have been searched) 94

#4 "Kallmann syndrome" (Word variations have been searched 10

#5 (#1)OR(#2)OR(#3)OR(#4) 367

#6 MeSH descriptor: [Reproductive Techniques, Assisted] explode all trees 3437

#7 MeSH descriptor: [Sperm Injections, Intracytoplasmic] explode all trees 591

#8 MeSH descriptor: [Fertilization in Vitro] explode all trees 2214

#9 assisted reproductive techniques (Word variations have been searched) 722

#10 intrauterine insemination (Word variations have been searched) 887

#11 intracytoplasmic sperm injection (Word variations have been searched) 1770

#12 testicular sperm extraction (Word variations have been searched) 67

#13 in vitro fertilization (Word variations have been searched) 3702

#14 embryo transplantation (Word variations have been searched) 58

#15 intra-fallopian transfer (Word variations have been searched) 32

#16 (#6)OR(#7)OR(#8)OR(#9)OR(#10)OR(#11)OR(#12)OR(#13)OR(#14)OR(#15) 6126

#17 (#5)AND(#16) 8

**Search strategy for Embase**

#1 'hypogonadotropic hypogonadism'/exp OR 'hypogonadotropic hypogonadism' 4973

#2 'kallmann syndrome'/exp OR 'kallmann syndrome' 1594

#3 (#1)OR(#2) 5766

#4 'assisted reproductive techniques'/exp OR 'assisted reproductive techniques' 107174

#5 'intrauterine insemination'/exp OR 'intrauterine insemination' 4828

#6 'intracytoplasmic sperm injection'/exp OR 'intracytoplasmic sperm injection'

19024

#7 'testicular sperm extraction'/exp OR 'testicular sperm extraction' 1919

#8 'in vitro fertilization'/exp OR 'in vitro fertilization' 85065

#9 'embryo transplantation'/exp OR 'embryo transplantation' 27317

#10 'intra-fallopian transfer'/exp OR 'intra-fallopian transfer' 220

#11 (#4)OR(#5)OR(#6)OR(#7)OR(#8)OR(#9)OR(#10) 110237

#12 (#3)AND(#11) 504
